# Effect of Cerium on Inclusion Modification in a Secondary-Hardening Steel

**DOI:** 10.3390/ma16113972

**Published:** 2023-05-25

**Authors:** Shun Han, Ruming Geng, Simin Lei, Yong Li, Chunxu Wang

**Affiliations:** Research Institute of Special Steels, Central Iron and Steel Research Institute, Bejing 100081, China; hanshunfa@126.com (S.H.); gengruming@nercast.com (R.G.); leisimin@nercast.com (S.L.); wangchunxu@nercast.com (C.W.)

**Keywords:** inclusion modification, cerium treatment, thermodynamic calculations, secondary-hardening steel

## Abstract

Owing to the continuous increasing of steel strength, mechanical properties including toughness and fatigue performance are becoming increasingly sensitive to inclusions in ultra-high strength steel. Rare-earth treatment is considered as an effective method to reduce the harmful effects of inclusions, but is rarely applied in secondary-hardening steel. In the present study, different amounts of cerium were added in a secondary-hardening steel to investigate the modification effect of Ce on non-metallic inclusions in steel. The characteristics of inclusions were observed experimentally using SEM-EDS and the modification mechanism was analyzed based on thermodynamic calculations. The results indicated that the main inclusions in Ce-free steel are Mg-Al-O + MgS. Thermodynamic calculation indicated that MgAl_2_O_4_ is firstly formed in liquid steel and then successively transformed into MgO and MgS during cooling process. When the Ce content is 0.0030%, the typical inclusions in steel were individual Ce_2_O_2_S and MgO + Ce_2_O_2_S complex inclusions. When the Ce content was increased to 0.0071%, the typical inclusions in steel were individual Ce_2_O_2_S- and Mg-containing inclusions. Ce treatment modifies the angular magnesium aluminum spinel inclusions into spherical and ellipsoidal Ce-containing inclusions, thus reducing the harmful effect of inclusion on steel properties.

## 1. Introduction

As the aviation, aerospace, military equipment, and other fields continue to develop, their requirement for high-performance structural materials is also significantly increased. Owing to its excellent combination of strength, toughness, and fatigue properties, secondary-hardening steel has become a key material in many load-bearing components, such as aircraft landing gears and engine shafts [1,2].

However, with the strength enhancement of steel, the damage caused by inclusions to the fatigue performance is also more serious. Because fatigue failure preferentially originates from inclusions, the characteristics of inclusions, including size, type, and morphology, obviously affected the fatigue performance of high-strength steel [3,4,5]. In recent decades, the development of metallurgical equipment and smelting technology has significantly improved the cleanliness of molten steels [6,7]. As commercial smelting processes of ultra-high-strength steel, vacuum induction melting and vacuum arc remelting are used to control the total oxygen (T.O) content of steel below 0.0010% [8,9]. However, it is difficult to further enhance steel cleanliness by improving metallurgical equipment in a short period. Rare-earth treatment is considered an effective method for modifying inclusions and reducing their harmful effects [10,11,12]. 

Wang et al. [13] investigated the effect of cerium (Ce) on the morphology of Al_2_O_3_ inclusions and determined that, after adding Ce, inclusions changed from a morphology of long strip and sharp angle to spherical and spindle surface. Zhang et al. [14] demonstrated the evolution path of inclusions with an increased Ce content in Si-Mn-killed steel, from Al_2_O_3_-SiO_2_-MnO-CaO to Ce_2_O_3_-Al_2_O_3_-SiO_2_-MnO-CaO, Ce_2_O_3_, and Ce_2_O_3_-CeS. Huang et al. [15] reported the inclusion evolution process when the Ce content increased from 0–0.03% in H13 steel, as changed from MgO·Al_2_O_3_ to CeAlO_3_, Ce-O, and Ce-O-S 

The modified rare-earth inclusions also played a crucial role in improving the properties of steel. Huang et al. [16] found that MnS and MnO-SiO_2_ inclusions were modified into (Ce,La)_2_O_2_S; a good compatibility between rare-earth inclusions and the matrix decreased the corrosion rate and improved the corrosion resistance. Ji et al. [17] summarized the beneficial role of rare-earth treatment on grain refinement, indicating that rare-earth inclusions acted as a nucleation core to promote the formation of the δ-Fe (BCC structure) and γ-Fe (FCC structure) in liquid steel, refining solidification microstructures and reducing element segregation. Yang et al. [18,19] indicated that the rare-earth addition modified the (Ca,Mn)S inclusion into a rare-earth inclusion in bearing steel, prolonging the fatigue life over ten times. The beneficial effects of rare-earth inclusions on welding performance has also been reported [20,21].

However, although rare-earth treatment has been applied to many types of steels, including bearing steel [22], high-speed steel [23], die steel [24], etc., its application in secondary-hardening steel was rarely reported. In addition, there is also little research on the inclusion evolution in rare-earth steel containing high contents of Mg and Al. Therefore, the effect of cerium on inclusion modification in a secondary-hardening steel with a high Al content was investigated in the present study. Ingots with different Ce contents were smelted and casted, the characteristics of inclusions were observed experimentally, and the modification mechanism was analyzed based on thermodynamic calculations.

## 2. Materials and Methods

The experimental steels were prepared by a 25 kg vacuum arc melting furnace in this study. To ensure high cleanliness of experimental steels, raw materials with low impurity contents was melted in a MgO crucible at 1550 °C. After melting, the liquid steel was cast into a shell ingot, designated ingot S1. For comparing Ce modification on inclusions, pure Ce alloy was added into liquid steel after deoxidation process. For comparison, two ingots containing different rare-earth contents were manufactured, designated as S2 and S3, respectively. 

Each ingot was machined into steel powders to analyze the Ca, Mg, and Ce contents using inductively coupled plasma spectrometry ((NCS Plasma MS 300, NCS Testing Technology Corporation, Beijing, China). The T.O and N contents were determined using an O, N, and H analyzer (NCS ONH-5500, NCS Testing Technology Corporation, Beijing, China). The C and S contents were measured using a C and S analyzer (NCS CS-3000, NCS Testing Technology Corporation, Beijing, China). The contents of other elements were tested using an optical emission spectrometer (NCS SparkCCD 7000, NCS Testing Technology Corporation, Beijing, China). Table 1 gives the chemical compositions of the experimental steels. The non-metallic inclusions in the samples were observed using a scanning electron microscope (SEM, EVO LS 25, ZEISS Corporation, Oberkochen Germany), and their compositions were determined using an energy-dispersive spectrometer (EDS) equipped with an acceleration voltage of 20 kV.

## 3. Results and Discussion

### 3.1. Inclusions in Ce-Free Steel

**Figure 1:**
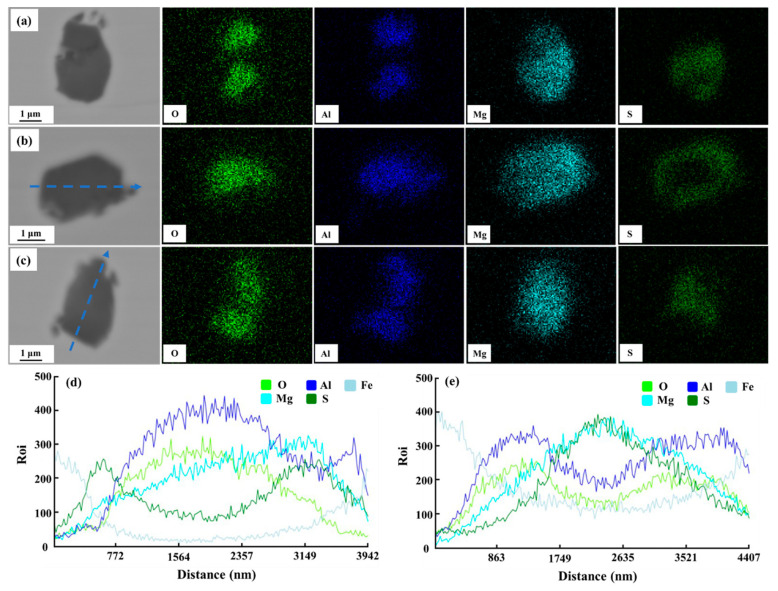
Typical inclusions observed in sample S1: (**a**–**c**) elements mappings, (**d**,**e**) line scanning results of the blue lines marked in (**b**,**c**), respectively.

Figure 1 presents the backscattered electron images (BSE) and EDS results of the typical inclusions in sample S1. From the element mappings shown in Figure 1a–c, it was concluded that the distributions of Al and O showed a good consistency, in which a little Mg was also present. Moreover, Mg was also present in areas containing S. The same conclusion could also be drawn from the line-scanning results as shown in Figure 1d,e. The typical inclusions in the sample without a Ce addition could be identified as Mg-Al-O + MgS based on the SEM-EDS determination.

Due to a strong binding ability with O, Al_2_O_3_ inclusion is a common deoxidation product in Al-containing steel. Soluble Mg came from the refractory materials during the vacuum arc melting process, and can react with Al_2_O_3_ inclusions to form an Mg-Al-O inclusion [25,26]. With the increasing Al content, the inclusions gradually transformed from Al_2_O_3_ into MgO·Al_2_O_3_ after Mg treatment, and the mole ratio of MgO/Al_2_O_3_ in the inclusions decreased [27].

### 3.2. Inclusions in Steel Containing 0.0030% Ce

**Figure 2:**
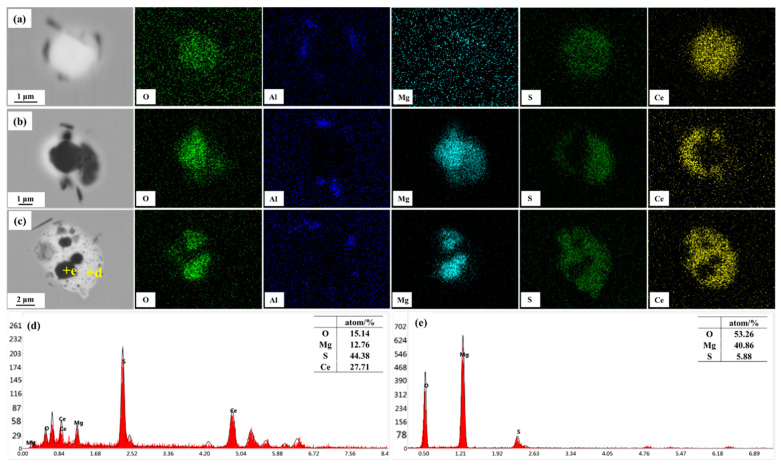
Typical inclusions observed in sample S2: (**a**–**c**) elements mappings, (**d**,**e**) EDS analysis of the points marked in (**c**).

In recent years, rare-earth treatment to modify inclusions in steel have been extensively studied [28,29], to reduce their harmful effects and even play a beneficial role in some fields, such as oxide metallurgy [30]. Figure 2 presents the typical inclusions in the steel containing 0.0030% Ce.

From SEM-EDS analysis, the typical inclusions in sample S2 can be classified into two types. The first was an individual Ce-containing inclusion, which could be identified as Ce_2_O_2_S (Figure 2a). The other was the MgO + Ce_2_O_2_S complex inclusion, in which MgO as a black core was surrounded by Ce_2_O_2_S (Figure 2b,c). This morphology of the MgO + Ce_2_O_2_S complex inclusion was mainly influenced by the modification mechanism of Ce on MgO inclusion, which will be analyzed in detail later.

### 3.3. Inclusions in Steel Containing 0.0071% Ce

**Figure 3:**
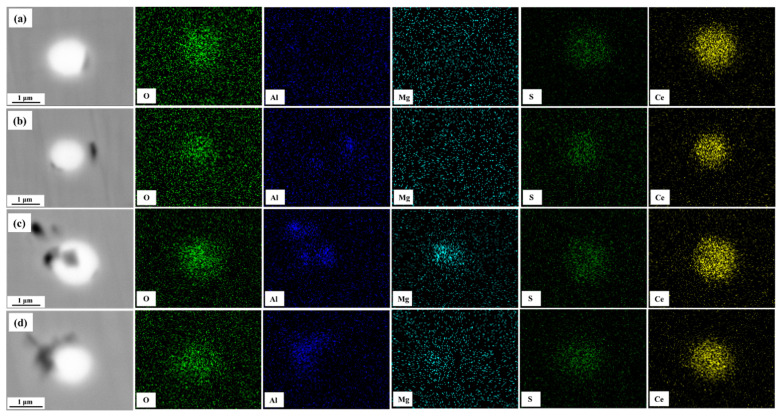
Characteristics of rare-earth inclusions observed in sample S3. (**a**–**d**) elements mappings of rare-earth inclusions.

**Figure 4:**
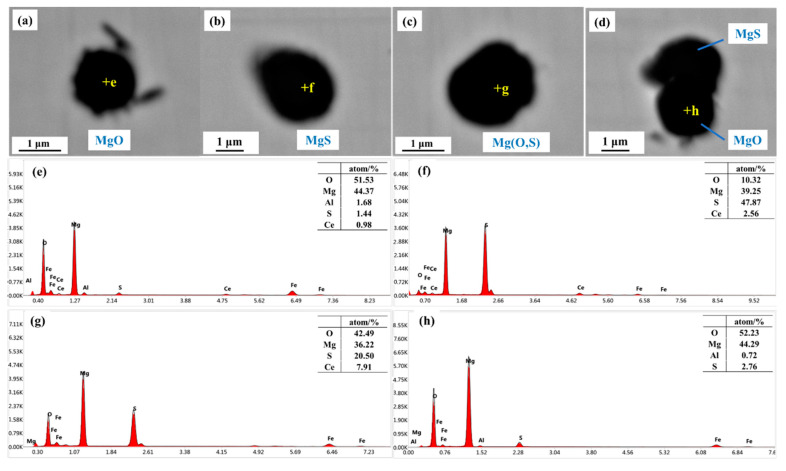
Individual Mg-containing inclusions observed in sample S3: (**a**–**d**) SEM photos of inclusions, (**e**–**h**) EDS analysis of the points marked in (**a**–**d**), respectively.

Figure 3 presents the element mappings of the rare-earth inclusions in sample S3. When the Ce content increased to 0.0071%, many individual rare-earth inclusions were formed in the sample based on the SEM-EDS determination results. The elemental mapping results indicated that the rare-earth inclusions were Ce_2_O_2_S. In addition, many individual Mg-containing inclusions were observed in sample S3, as shown in Figure 4, and were mainly composed of MgO, MgS, and Mg(O,S). In addition, Figure 3a–d exhibited that Ce_2_O_2_S inclusions were smaller than the inclusions in sample S1, and were basically spherical.

### 3.4. Thermodynamic Calculation of Magnesium Aluminum Spinels

**Figure 5:**
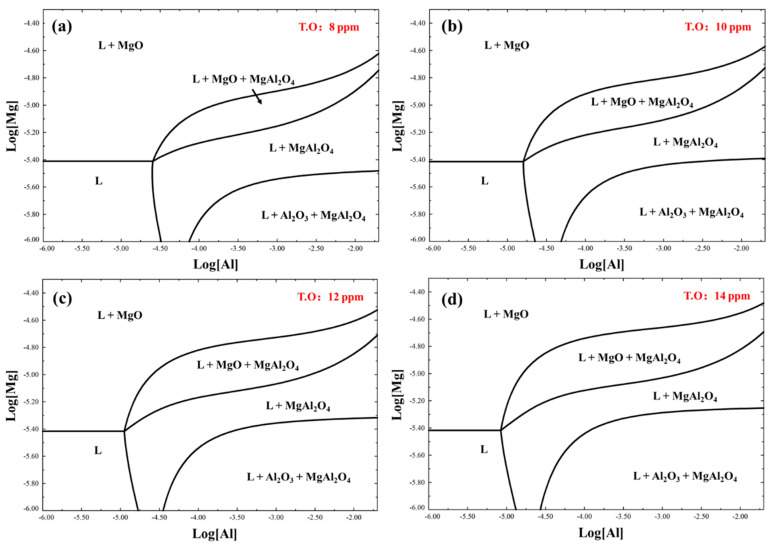
Inclusion-forming region of Fe−O−S−Mg−Al system at 1550 °C: (**a**) T.O content is 8 ppm, (**b**) T.O content is 10 ppm, (**c**) T.O content is 12 ppm, (**d**) T.O content is 14 ppm.

The formation of magnesium aluminum spinel inclusions was related to the contents of Mg, Al, O, and S. Figure 5 gives the inclusion-forming region of Fe-O-S-Mg-Al system at 1550 °C, calculated using FactSage 8.0. Based on the chemical compositions listed in Table 1, the databases of FSstel, FactPA and FToxid were used in the calculation. The Mg and Al contents varied from 0.0001–0.0050% and 0.0001–2%, respectively. In addition, different T.O contents from 0.0008–0.0014% were also considered in the calculation. Figure 5a shows that when the Al content was more than 0.0030%, magnesium aluminum spinel inclusions could be formed in the liquid steel, even at an Mg content of as low as 1 ppm. At the same Al content, such as that containing 0.01% Al, as the Mg content increased, the inclusion-forming region gradually changed from Al_2_O_3_ + MgAl_2_O_4_ to MgAl_2_O_4_ and MgO. The stability diagram under different oxygen contents indicated that a higher oxygen content obviously enhanced the region of L + MgO + MgAl_2_O_4_ and L + Al_2_O_3_ + MgAl_2_O_4_, while the forming region of MgO is reduced.

**Figure 6:**
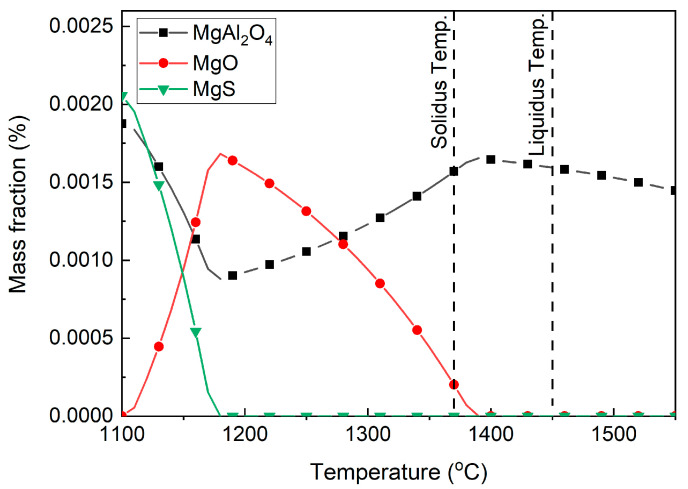
Inclusion transformation in sample S1 from 1100 °C to 1550 °C.

To further investigate the inclusion evolution in sample S1, the inclusion transformation from 1100 °C to 1550 °C was analyzed, with a calculation step size of 10 °C, as shown in Figure 6. The liquidus and solidus temperatures of the experimental steel were calculated as 1450 °C and 1370 °C, respectively, and marked using dashed lines. MgAl_2_O_4_ was firstly formed in liquid steel at 1550 °C. With the temperature decreased to 1380 °C, MgO gradually formed, accompanied by a decrease in MgAl_2_O_4_. This reaction can be expressed as Equation (1) [31,32]. A high Mg content can promote this reaction.

(1) $${\mathrm{MgAl}}_{2}O_{4}(s)+3[Mg]=4MgO(s)+2[Al]$$

Then, MgO transformed into MgS and the amount of MgAl_2_O_4_ increased, at temperatures lower than 1180 °C. This result was consistent with the inclusions through experimental observation.

### 3.5. The Effect of Ce on Oxide Inclusions

**Figure 7:**
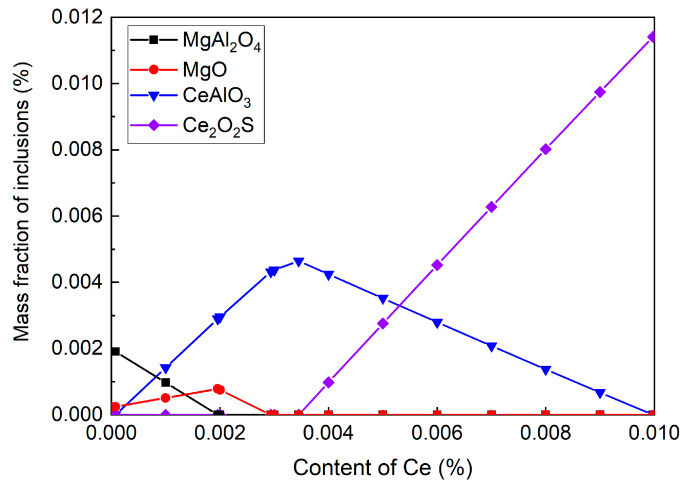
Inclusion transformation with different Ce contents at 1550 °C.

Figure 7 shows the inclusion transformation with different Ce contents at 1550 °C, wherein the chemical composition of sample S2 was used. It could be concluded that MgAl_2_O_4_ was formed at molten steel without adding Ce. With the increasing Ce content, the amount of MgAl_2_O_4_ decreased, and MgO was gradually formed. When the Ce content reached 0.0007%, CeAlO_3_ was formed in steel and its amount gradually increased with the increasing Ce contents. The maximum mass fraction of CeAlO_3_ was obtained in molten steel containing 0.0035% Ce. Further increasing the Ce content, CeAlO_3_ inclusion was transformed into Ce_2_O_2_S. The thermodynamic calculations showed that the inclusion transformation path was MgAl_2_O_4_→CeAlO_3_→Ce_2_O_2_S.

**Figure 8:**
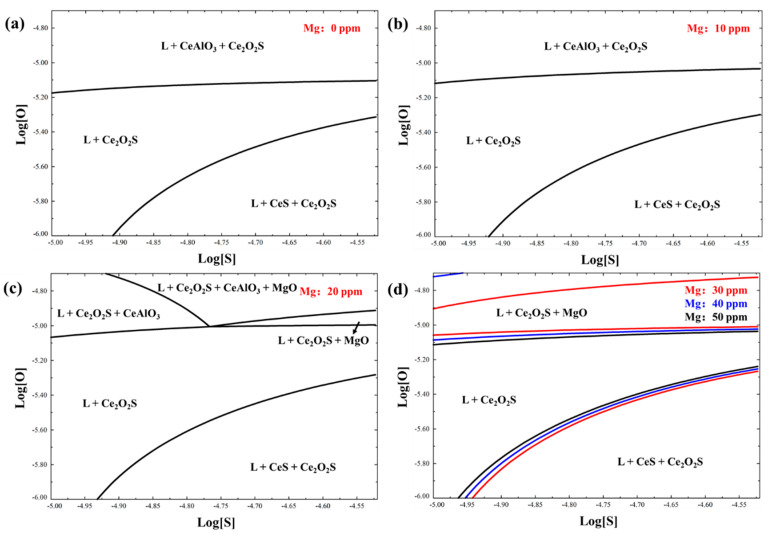
Rare-earth-inclusion-forming region of Fe−Ce−O−S−Mg−Al system at 1550 °C: (**a**) Mg content is 0 ppm, (**b**) Mg content is 10 ppm, (**c**) Mg content is 20 ppm, (**d**) Mg contents are 30, 40 and 50 ppm.

However, rare-earth aluminates were directly transformed into rare-earth oxide sulfides in this calculation, while no rare-earth oxides were formed. This result was slightly different from other reports [15,28], which may be influenced by the high Mg content. Thus, the rare-earth inclusion forming region with different Mg contents from 0 to 0.0050% at 1550 °C was calculated (Figure 8). The T.O and S contents varied from 0.0001–0.0020% and 0.0010–0.0030%, respectively, set as the vertical and horizontal co-ordinates of the figure. In addition, the Ce and Al contents were fixed as 0.0070% and 0.01%, respectively.

Figure 8a indicated that when the Mg content was 0, the typical rare-earth inclusions at 1550 °C were CeAlO_3_, Ce_2_O_3_, and Ce_2_O_2_S. When the Mg content was increased to 0.0020%, the stability region of MgO was formed. Figure 8d demonstrated that, by further increasing the Mg content to 0.0030%, 0.0040%, and 0.0050%, the formation region of Ce-containing sulfides and MgO were enlarged. Li et al. [33] reported similar results and indicated that the activity interaction coefficients $e_{O}^{\mathrm{Mg}}$ and $e_{S}^{\mathrm{Mg}}$ in molten steel at 1600 °C were −300 and −1.82, respectively. Thus, the generation of rare-earth oxides was reduced and that of rare-earth sulfides was promoted by the addition of Mg.

**Figure 9:**
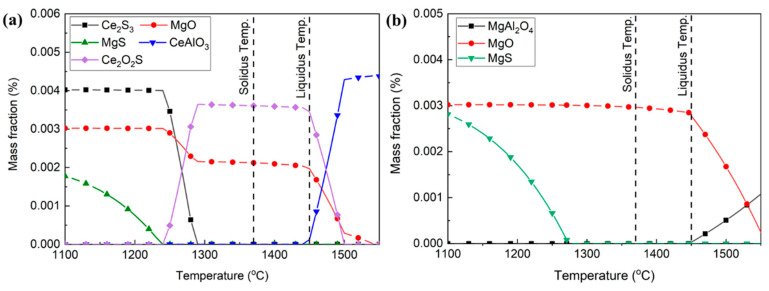
Inclusion transformation in sample S2 from 1100 °C to 1550 °C: (**a**) containing 0.0030% Ce, (**b**) neglecting the effect of Ce.

Figure 9a shows the inclusion transformation in sample S2 from 1100 °C to 1550 °C. Containing 0.0030% Ce, CeAlO_3_ was formed in liquid steel at 1550 °C, the remelting temperature. MgO was precipitated at 1540 °C. When the temperature was cooled to 1490 °C, CeAlO_3_ inclusions were transformed into Ce_2_O_2_S, which could be expressed as Equation (2) [28].

(2) $${\mathrm{CeAlO}}_{3}(s)+2[Ce]+3/2[S]=3/2Ce_{2}O_{2}S(s)+[Al]$$

When the temperature further decreased to 1280 °C, Ce_2_O_2_S furtherly transformed into Ce_2_S_3_ inclusions. Ren et al. [34] has reported this transformation in ultra-low-carbon aluminum-killed steel, as shown in Equation (3).

(3) $${\mathrm{Ce}}_{2}O_{2}S(s)+4/3[Al]+2[S]=2/3Al_{2}O_{3}(s)+Ce_{2}S_{3}(s)$$

However, in the present study, the experimental steel had a high Mg content. The O in Ce_2_O_2_S combined with soluble Mg in steel to produce MgO instead of the formation of Al_2_O_3_, which was obtained from the calculation as the increase in the amount of MgO. Thus, this reaction in the present study should be expressed as Equation (4).

(4) $${\mathrm{Ce}}_{2}O_{2}S(s)+2[Mg]+2[S]=2MgO(s)+Ce_{2}S_{3}(s)$$

The morphology of inclusions after the Ce addition was closely related to the inclusion before adding Ce. Therefore, Figure 9b also presents the inclusion precipitation while neglecting the effect of Ce. Furthermore, MgO was the predominant inclusion at 1550 °C. Thus, after the Ce addition, Ce firstly reacted with MgO in molten steel to form Ce_2_O_2_S. Moreover, Figure 2b,c exhibited a morphology of an unreacted core of the Mg-containing inclusion surrounded by an outer Ce-containing inclusion, which represents an incomplete modification of Ce treatment, and this process could be explained using the unreacted core model [35,36].

**Figure 10:**
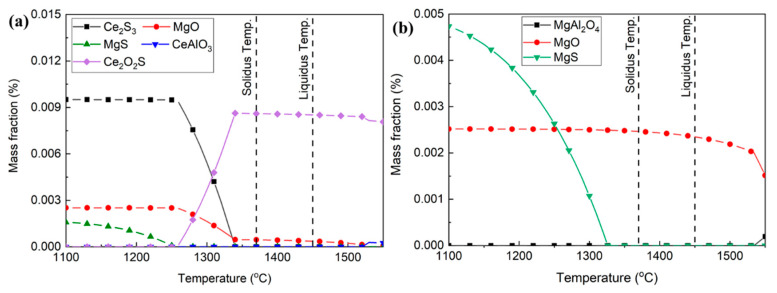
Inclusion transformation in sample S3 from 1100 °C to 1550 °C: (**a**) containing 0.0071% Ce, (**b**) neglecting the effect of Ce.

The inclusion transformation in sample S3 was also calculated by considering the effect of Ce and neglecting its effect, as exhibited in Figure 10a,b, respectively. Before the Ce addition, the typical inclusions in sample S3 were MgO. When the Ce content was 0.0071%, the typical inclusions in molten steel were Ce_2_O_2_S. Then, Ce_2_O_2_S was transformed into Ce_2_S_3_ and MgO as the temperature was lower than 1330 °C.

## 4. Conclusions

In this study, the effect of Ce on inclusion modification in a secondary-hardening steel was investigated. The characteristics of inclusions in Ce-free and Ce-containing steel were contrastively studied based on experimental determinations and thermodynamic calculations. The following conclusions are obtained:

1. For the steel considered herein, typical inclusions in Ce-free steel were Mg-Al-O + MgS. Thermodynamic calculations indicated that MgAl_2_O_4_ was firstly formed in liquid steel, successively transforming into MgO and MgS during cooling.
2. When the Ce content was 0.0030%, the typical inclusions in steel were individual Ce_2_O_2_S and MgO + Ce_2_O_2_S complex inclusions. For the latter type of inclusion, MgO as a core was surrounded by an outer layer of Ce_2_O_2_S, for which the modification process could be explained through the unreacted core model. When the Ce content was 0.0071%, the steel mainly consisted of individual Ce_2_O_2_S- and Mg-containing inclusions. The latter included MgO, MgS, and Mg(O,S).
3. Ce treatment modified the angular magnesium aluminum spinel inclusions into spherical and ellipsoidal Ce-containing inclusions, which was beneficial for reducing the harmful effect of inclusion on steel properties. Thermodynamic calculations indicated that the generation of rare-earth oxides was reduced and that of rare-earth sulfides was promoted by the addition of Mg.

## Figures and Tables

**Table 1:** Chemical compositions of experiment steels (mass%).

Sample numbers	C	Cr	Ni	Mo	Al	Co
S1	0.23	2.3	14.00	1.5	1.00	10
S2	0.23	2.3	14.00	1.5	1.00	10
S3	0.23	2.3	14.00	1.5	1.00	10
Sample numbers	Ca	Mg	Ce	T.O	S	N
S1	<0.0005	0.0015	0	0.0013	0.0020	0.0010
S2	<0.0005	0.0032	0.0030	0.0012	0.0022	0.0009
S3	<0.0005	0.0037	0.0071	0.0010	0.0034	0.0009

## Data Availability

Not applicable.
